# Myosin Heavy Chain 7 (MYH7) Variant Associated Cardiovascular Disease: An Unusual Case of Heart Failure in a Young Male

**DOI:** 10.7759/cureus.61252

**Published:** 2024-05-28

**Authors:** Lekhini Latchupatula, Myles Benayon, Muqtasid Mansoor, Judy Luu

**Affiliations:** 1 Internal Medicine, McMaster University, Hamilton, CAN; 2 Medical School, McMaster University, Hamilton, CAN; 3 Cardiology, McGill University, Montreal, CAN

**Keywords:** cardiomyopathy, genetics, congenital, bicuspid aortic valve, myocarditis, excessive trabeculation, heart failure, myh7

## Abstract

A 37-year-old male with type two diabetes presented to the hospital with new-onset heart failure and renal dysfunction. His left ventricular (LV) ejection fraction was less than 10%. Transthoracic echocardiography and cardiovascular magnetic resonance (CMR) imaging also revealed severe bicuspid aortic valve stenosis, dilated cardiomyopathy with LV hypertrophy, prominent LV trabeculations, and features suggestive of mild myocarditis with active inflammation. While myocarditis was suspected on CMR imaging, his mild degree of myocardial involvement did not explain the entirety of his clinical presentation, degree of LV dysfunction, or other structural abnormalities. An extensive work-up for his LV dysfunction was unremarkable for ischemic, metabolic, infiltrative, infectious, toxic, oncologic, connective tissue, and autoimmune etiologies. Genetic testing was positive for a *myosin heavy chain 7* (*MYH7*) variant, which was deemed likely to be a unifying etiology underlying his presentation.

The *MYH7* sarcomere gene allows beta-myosin expression in heart ventricles, with variants associated with hypertrophic and dilated cardiomyopathies, congenital heart diseases, myocarditis, and excessive trabeculation (formerly known as left ventricular noncompaction). This case highlights the diverse array of cardiac pathologies that can present with *MYH7* gene variants and reviews an extensive work-up for this unusual presentation of heart failure in a young patient.

## Introduction

This article was previously presented as an abstract and poster at the 2023 Rocky Mountain Internal Medicine Conference on November 17, 2023.

The *myosin heavy chain 7* (*MYH7*) sarcomere gene codes for the β-cardiac myosin heavy chain within cardiomyocytes, an essential protein structure for cardiac contractility [1,2]. Excitation-contraction coupling is the mechanism that underpins cardiac inotropy, which is facilitated by myosin moving along actin filaments to create myocardial contraction [1]. *MYH7* gene mutations have been associated with dilated and hypertrophic cardiomyopathies, which can lead to complications such as heart failure and sudden cardiac death [1,3]. These same mutations have also been linked to cardiac pathologies such as excessive trabeculation (formerly known as left ventricular noncompaction), bicuspid aortic valves (BAV), and myocarditis [4-7].

Hypertrophic cardiomyopathies are characterized by the thickening of the left ventricle (LV), which primarily affects the interventricular septum and causes diastolic dysfunction [3]. In contrast, dilated cardiomyopathies are characterized by the enlargement of a ventricular chamber, which results in systolic dysfunction [3]. A BAV is a congenital heart defect in which the aortic valve has only two leaflets instead of the normal three leaflets [4].

Excessive trabeculation is described as a bi-layered myocardium of trabeculation and compacted myocardium [7]. This condition has been associated with LV hypertrophy, endomyocardial thickening, and deep intertrabecular recesses, which can variably present with adverse events such as heart failure, arrhythmias, and embolic events of both the venous and arterial systems [7]. Myocarditis is pathologic myocardial inflammation secondary to a wide array of infectious and non-infectious etiologies. Clinically, patients vary from being asymptomatic to having significant complications, including heart failure and conduction disease [8].

In this case report, we discuss an unusual presentation of new-onset heart failure in a 37-year-old male with an *MYH7* variant and features of excessive trabeculation, congenital heart disease, and myocarditis.

## Case presentation

A 37-year-old male with a past medical history of type 2 diabetes, depression, anxiety, and asthma presented to his local hospital with a two-week history of worsening dyspnea. Upon further prompting, the patient endorsed orthopnea and paroxysmal nocturnal dyspnea. He reported no recent infections. On examination, his oxygen saturation was 95% on 2 L via nasal cannula, and he was afebrile. He was tachycardic at 108 beats per minute, hypertensive at 159/117 mmHg, and tachypneic at 24 breaths per minute. Auscultation revealed a right upper sternal border systolic ejection murmur and bilateral basilar crackles with a diffuse wheeze. Significant lower extremity bilateral pitting edema was present, extending to his knees with an elevated jugular venous pressure. 

The patient had no significant family history related to cardiac disease or otherwise. He had no known allergies. His routine outpatient medications included escitalopram, fluticasone-salmeterol, ipratropium bromide, metformin, quetiapine, rabeprazole sodium, sitagliptin, and zopiclone. The patient is a life-long non-smoker of tobacco cigarettes but smokes marijuana daily.

Blood tests (Table 1) were significant for a troponin elevation of 53 ng/L (reference value: ≤ 34 ng/L) and creatinine elevation of 298 µmol/L (reference value: 60-110 µmol/L). His troponin and creatinine peaked at 59 ng/L and 416 µmol/L, respectively. His electrocardiogram (ECG) revealed sinus tachycardia without specific evidence of LV hypertrophy. Serial ECGs showed no dynamic changes that would be concerning for acute ischemia. A coronary angiogram revealed normal coronary arteries. His initial transthoracic echocardiogram (TTE) revealed an LV ejection fraction (LVEF) of less than 10% (Video 1), which was also demonstrated using an ultrasound-enhancing contrast agent (Video 2). Other findings included low-flow, low-gradient severe aortic stenosis (AS), BAV, severe LV dilatation, and severely increased ventricular mass and wall thickness. 

**Table 1:** Summary of laboratory values throughout hospital admission.

Lab Test	Result	Reference Range
Cardiac and Renal Profile		
Angiotensin-Converting Enzyme (U/L)	22	16-85
Creatinine (µmol/L) - Initial	298	60-110
Creatinine (µmol/L) – Peak	416	60-110
Estimated Glomerular Filtration Rate (ml/min/1.73m2)	23	>60
Troponin l-high-sensitivity (ng/L) - Initial	53	0-34
Troponin l-high-sensitivity (ng/L) - Peak	59	0-34
Complete Blood Count and Cell Differential		
Hemoglobin (g/L)	113	130-180
Leukocytes (x10^9^/L)	11.9	4-11
Mean Corpuscular Volume (fL)	90.7	82-99
Platelets (x10^9^/L)	294	150-400
Absolute Neutrophil Count (x10^9^/L)	9.8	2-7.5
Basophils (x10^9^/L)	0.1	0-0.1
Eosinophils (x10^9^/L)	0.1	0-0.4
Lymphocytes (x10^9^/L)	1	1.5-4
Monocytes (x10^9^/L)	1	0.2-0.8
Electrolytes		
Chloride (mmol/L)	103	95-110
Ionized Calcium (mmol/L)	1.19	1.16-1.29
Magnesium (mmol/L)	0.84	0.7-1.1
Phosphate (mmol/L)	1.59	0.9-1.52
Potassium (mmol/L)	5.4	3.5-5.2
Sodium (mmol/L)	134	135-145
Liver Enzymes and Function Tests		
Alanine Transaminase (U/L)	97	0-49
Alkaline Phosphatase (U/L)	84	38-126
Bilirubin (µmol/L)	10	0-20
Gamma-Glutamyl Transferase (U/L)	163	0-64
International Normalized Ratio	1.3	0.8-1.1
Immune and Inflammatory Markers		
Anti-Glomerular Basement Membrane Antibody (Al)	<0.2	0-0.9
Antineutrophil Cytoplasmic Antibody (Al Al)	<0.2	0-0.9
Antinuclear Antibody	Negative	Not Applicable
Complement C3 (g/L)	0.98	0.79-1.52
Complement C4 (g/L)	0.15	0.16-0.38
C-Reactive Protein (mg/L)	21.3	0-9
Cryoglobulin	Negative	Not Applicable
Immunoglobulin G4 (g/L)	0.205	0.039-0.864
Perinuclear Anti-Neutrophil Cytoplasmic Antibody (Al Al)	<0.2	0-0.9
Rheumatoid Factor (IU/mL)	<20	0-19
Serum Protein Electrophoresis		
Albumin Electrophoresis (g/L)	42	35-52
Alpha 1 Globulin (g/L)	2	1-4
Alpha 2 Globulin (g/L)	7	4-10
Beta Globulin (g/L)	6	5-12
Gamma Globulin (g/L)	10	6-16
Urine Protein Electrophoresis		
Albumin, Urine (g/d)	3.77	Not Applicable
Globulin, Urine (g/d)	0.98	Not Applicable
Protein, Urine (24-hour) (g/d)	4.75	0-0.14
Miscellaneous		
Ferritin (ug/L)	49	Not Applicable
Kappa/Lambda Light Chain Ratio	1.14	0.26-1.65
Reticulocytes (x10^9^/L)	115	10-86
Thyroid Stimulating Hormone (mlU/L)	0.62	0.47-4.68
Total Iron Binding Capacity (µmol/L)	58	40-80
Microbiology		
Adenovirus DNA	Negative	Not Applicable
COVID-19 RNA	Negative	Not Applicable
Hepatitis B Core Antibody and Surface Antibody/Antigen	Non-Reactive	Not Applicable
Hepatitis C antibody	Non-Reactive	Not Applicable
Human Immunodeficiency Virus	Non-Reactive	Not Applicable
Influenza A and B RNA	Negative	Not Applicable
Parainfluenza 3 RNA	Negative	Not Applicable
Rhino/Enterovirus RNA	Negative	Not Applicable
Respiratory Syncytial Virus RNA	Negative	Not Applicable

**Video 1:** Initial admission transthoracic echocardiogram in apical four-chamber view showing a reduced left ventricular ejection fraction of less than 10% calculated by two-dimensional Simpson biplane and a dilated left ventricle.

**Video 2:** Initial admission transthoracic echocardiogram demonstrating a reduced left ventricular ejection fraction of less than 10% using an ultrasound-enhancing contrast agent.

**Figure 1:**
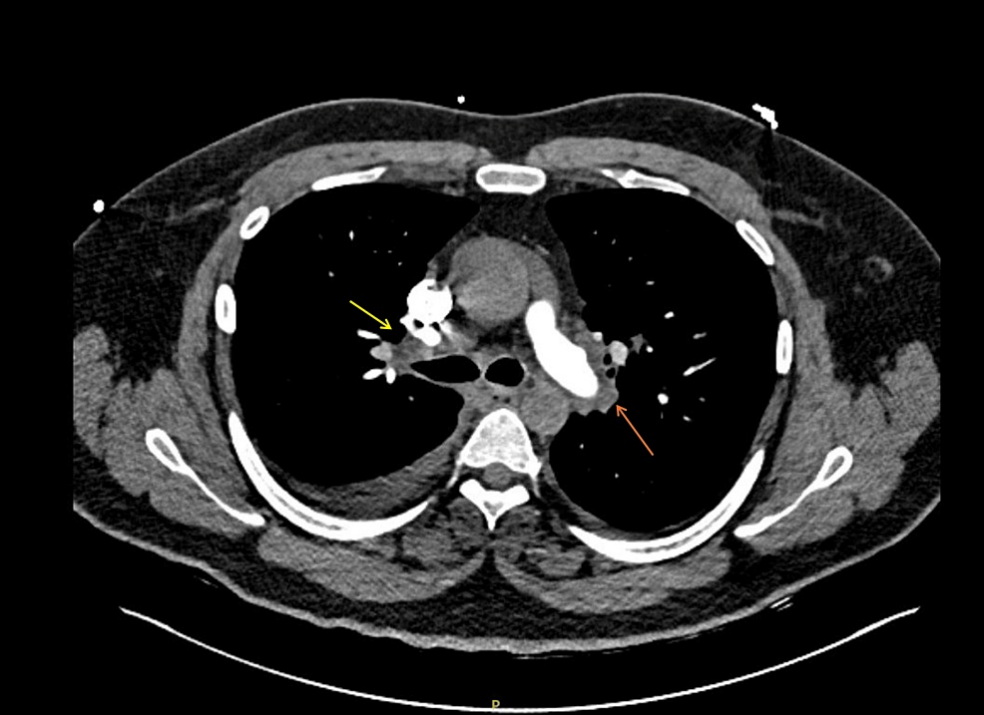
Computed tomography of the chest depicting enlarged mediastinal (yellow arrow) and hilar (orange arrow) lymph nodes.

A computed tomography (Figure 1) scan of his chest was ordered given his hypoxia. This was negative for a pulmonary embolism but revealed hilar lymph node enlargement, which prompted an initial concern for sarcoidosis, and a temporary course of oral steroids was initiated for three consecutive days in total. This was later confirmed not to be the case based on an unremarkable sarcoidosis positron emission tomography nuclear scan and a negative bronchial biopsy that were both done several weeks later. Lastly, a renal biopsy showed focal and global glomerulosclerosis without granulomatous changes.

The patient was diagnosed with non-ischemic cardiomyopathy, an exacerbation of new-onset heart failure with reduced ejection fraction (HFrEF), and a cardiorenal acute kidney injury. He was treated with intravenous furosemide for diuresis. Intravenous diuretics continued for two weeks until transitioning to oral furosemide. In addition, the patient was started on isosorbide dinitrate, hydralazine, and spironolactone during this time period. The patient’s renal function limited the advancement of guideline-directed medical therapy for HFrEF.

**Figure 2:**
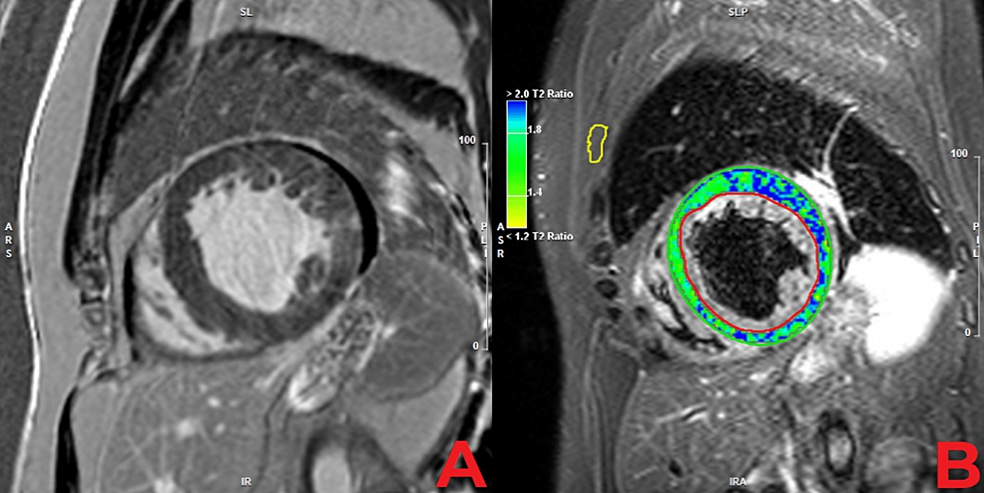
Cardiac magnetic resonance imaging showing a small area of hazy, subepicardial late gadolinium enhancement in the mid-inferolateral segment, adjacent to a pocket of effusion (Panel A) with concurrent elevated signal intensity on T2-weighted short tau inversion recovery images (Panel B), suggestive of mild myocarditis with active inflammation. There is also evidence of prominent left ventricular trabeculations.

Two weeks into his admission, a repeat TTE revealed an improved LVEF of 35%. Cardiovascular magnetic resonance imaging (CMR) was done approximately two weeks after the brief course of oral steroids described above. It showed a small area of hazy, subepicardial late gadolinium enhancement (LGE) in the mid-inferolateral segment with an adjacent pocket of effusion. His CMR imaging also revealed prominent LV trabeculations. His LGE findings with concurrent elevated signal intensity on T2-weighted short tau inversion recovery images suggested mild myocarditis with active inflammation (Figure 2). The consensus among his multiple cardiology specialists determined that the patient’s degree of myocardial involvement on LGE would not explain the entirety of his clinical presentation in florid heart failure, the degree of his LV impairment, or other structural abnormalities. The CMR findings were also not consistent with cardiac sarcoidosis. His extensive work-up was unremarkable for ischemic, metabolic, infiltrate, infectious, toxic, oncologic, connective tissue, and autoimmune etiologies. The patient was then discharged in stable condition with outpatient cardiology follow-up.

At the one-year follow-up, the patient’s LVEF recovered to 54% with optimized guideline-directed medical therapy on bisoprolol, dapagliflozin, hydralazine, and isosorbide dinitrate. Further guideline-directed medical therapy at that time was limited by the development of chronic kidney disease secondary to diabetic nephropathy. His creatinine and estimated glomerular filtration rate were 327 µmol/L and 20 mL/min/1.73 m^2^, respectively.

Outpatient genetic testing was done using the patient’s blood sample. Exome sequencing demonstrated positivity for an *MYH7* variant (c.1956+1G>A), and cardiogenetics consultation suggested this as a contributor to his non-ischemic cardiomyopathy. Further cardiology assessments concluded that urgent aortic valve replacement for his severe AS was not indicated, given symptom improvement. Given the absence of other clear causes, the unifying etiology of his cardiomyopathy and other structural cardiac abnormalities were deemed to be likely related to his *MYH7* variant.

## Discussion

Pathological chromosome 14 mutations of the *MYH7* gene can have deleterious effects on the β-cardiac myosin heavy chain structure, leading to dilated and hypertrophic cardiomyopathies and impairment of cardiac inotropy [1,2,9]. This case contributes to the growing body of evidence linking *MYH7* mutations not only to cardiomyopathies but also to a broader spectrum of cardiac pathologies, including myocarditis and structural heart diseases, such as BAV and excessive trabeculation.

The diagnosis of excessive trabeculation is increasingly being recognized through the use of CMR. The diagnostic criteria by Petersen et al. for excessive trabeculation is widely endorsed and requires the presence of (i) two distinct myocardial layers comprising a trabecular endocardial layer and compacted epicardial layer, (ii) endocardial trabeculations and profound intratrabecular recesses, and (iii) an end-diastolic trabecular to compacted ratio of ≥2.3:1 [7,10,11]. While this patient’s case does not meet the complete criteria for excessive trabeculation, he does show significant features on CMR, such as prominent LV trabeculations and LV hypertrophy [7,10].

*MYH7* is the most common mutated gene in patients with excessive trabeculation and can be simultaneously associated with Ebstein anomaly or other forms of congenital heart disease, such as BAV and aortic coarctation [4,5,7]. Furthermore, literature has described a definitive association between *MYH7* gene mutations and excessive trabeculation [7,12]. BAV is present in approximately one to two percent of the population and can lead to valvular abnormalities such as AS and aortic regurgitation [13].

Mutations in *MYH7* have been associated with myocarditis and are among the most represented in biopsy-proven myocarditis patients [6,14]. Genetic sequencing of adult patients with acute myocarditis also revealed an association with *MYH7* gene mutations [15]. 

On imaging, myocarditis in the acute phase can present with myocardial edema, chamber dilatation, pericardial effusions, and ventricular dysfunction. Weeks after the disease onset, myocardial edema tends to decline, but myocardial involvement within the lateral wall can persist in post-inflammatory myocarditis [16]. In this case report, the patient’s CMR findings of non-ischemic subepicardial LGE in the inferolateral walls with concurrent elevated T2 signal intensity suggested mild myocarditis with active inflammation of an unclear etiology [16]. However, expert cardiology consensus concluded that his degree of myocardial involvement would be too mild to explain the severe impairment in his LV dysfunction and would not explain his other pathological structural changes [16]. 

This patient’s dilated cardiomyopathy with LV hypertrophy, BAV, and features of myocarditis and excessive trabeculation are all conditions associated with pathogenic *MYH7* variants [1,3-7]. As per the clinical molecular geneticist analyzing this patient’s *MYH7* variant (c.1956+1G>A), "this occurred in the invariant region of the splice consensus region, and it is predicted to abolish or destroy the donor splice site of intron 16, which is expected to disrupt messenger ribonucleic acid splicing and likely result in an absent or disrupted protein (β-myosin in cardiomyocytes)." The gene zygosity was heterogenous, and the mode of inheritance was autosomal dominant. Given this genetic analysis and the absence of evidence for other etiologies after an extensive work-up, his *MYH7* variant is likely a unifying underlying contributor to his presentation. His practitioners in the cardiology and heart function clinics further suspected this as a predisposing condition.

Emerging pharmacological methods targeting the pathogenic effects of genetic cardiomyopathy variants are increasingly being explored and brought into clinical practice [17]. For instance, certain *MYH7* variants destabilize myosin's role in cardiac inotropy [17]. Through studying the biochemical effects of mutations such as these, the drug mavacamten was developed for patients with hypertrophic cardiomyopathy [17,18]. This drug is a small-molecule allosteric recently studied in the EXPLORER-HCM randomized control trial and improved LV outflow tract obstruction, exercise capacity, and health status in hypertrophic cardiomyopathy patients [19,20]. That is one example of why continued reporting on *MYH7* variants is essential to understanding its associated diverse cardiac pathologies. Future studies evaluating the penetrance of *MYH7* variants may allow for tailored patient care and elucidate the utility of genetic counseling for family members, treatments, and screening protocols.

## Conclusions

This case demonstrates the importance of investigating *MYH7* gene variants and their role in a wide range of cardiac pathologies, such as cardiomyopathies, heart failure, congenital heart diseases, excessive trabeculation, and myocarditis. This patient’s *MYH7* pathological variant as an underlying explanation for his presenting heart failure emphasizes the importance of considering genetic causes in atypical cardiac presentations. Genetic analysis is warranted, especially after an extensive initial non-diagnostic workup has been completed, as in this case. Future research into the penetrance of pathologic gene mutations is necessary to clarify the role of genetic counseling for relatives, inform the creation of novel interventions, and establish screening procedures.
